# Advancements in the Development of Anti-SARS-CoV-2 Therapeutics

**DOI:** 10.3390/ijms251910820

**Published:** 2024-10-09

**Authors:** Junjie Huang, Qianqian Ma, Zhengding Su, Xiyao Cheng

**Affiliations:** 1 Institute of Modern Fermentation Engineering and Future Foods, School of Light Industry and Food Engineering, Guangxi University, No. 100, Daxuedong Road, Nanning 530004, China; 2216301016@st.gxu.edu.cn; 2 School of Pharmaceutical Sciences and Institute of Materia Medica, Xinjiang University, Urumqi 830017, China; 102000581@hbut.edu.cn

**Keywords:** SARS-CoV-2, COVID-19, Omicron, drug development

## Abstract

Severe acute respiratory syndrome coronavirus 2 (SARS-CoV-2) is the virus that causes COVID-19, and so far, it has occurred five noteworthy variants of concern (VOC). SARS-CoV-2 invades cells by contacting its Spike (S) protein to its receptor on the host cell, angiotensin-converting enzyme 2 (ACE2). However, the high frequency of mutations in the S protein has limited the effectiveness of existing drugs against SARS-CoV-2 variants, particularly the Omicron variant. Therefore, it is critical to develop drugs that have highly effective antiviral activity against both SARS-CoV-2 and its variants in the future. This review provides an overview of the mechanism of SARS-CoV-2 infection and the current progress on anti-SARS-CoV-2 drugs.

## 1. Introduction

Severe acute respiratory syndrome coronavirus 2 (SARS-CoV-2), commonly referred to as COVID-19, has caused an unparalleled mark on the global public health infrastructure and economy [1]. Individuals infected with SARS-CoV-2 may manifest a spectrum of symptoms, spanning from mild fever, dry cough, and fatigue to severe respiratory ailments, pneumonia, and multi-organ dysfunction [2]. SARS-CoV-2 is a single-stranded RNA virus, belonging to the *Betacoronavirus* genus within the *Coronaviridae* family, with approximately 80% homology in genome sequence with SARS-CoV [3]. Both coronaviruses invade host cells depending on the targeted interaction between the receptor-binding domain (RBD) in the spike glycoprotein (S protein) and the human angiotensin-converting enzyme 2 (ACE2) [4].

Rapid mutation rate and potent infectivity, along with the persistent absence of efficacious-specific drugs for prevention and treatment, have compounded the challenge posted by the emergence of highly pathogenic and immune-evasive strains, such as Alpha (B.1.1.7), Beta (B.1.351), Gamma (P.1), Delta (B.1.617.2), and Omicron (B.1.1.529). These factors have perpetuated the COVID-19 pandemic, leading to substantial healthcare expenditures worldwide [5,6]. As the best way to prevent and treat COVID-19, more than 10 billion doses have been administered globally, providing humanity with a temporary line of defense. Although vaccination has significantly reduced severe cases and mortality, the need for potential COVID-19 therapeutics will be urgently replaced in the face of the loss of vaccine efficacy due to viral variation and its impact on specific populations. A discussion of potential therapeutics for COVID-19 in the context of the current vaccination campaign is therefore warranted. It is an important research strategy to clarify the main mechanism of SARS-CoV-2 invasion, identify the key molecules involved in this process as the targets for drug intervention, and use drugs (including small molecules, peptides, monoclonal antibodies, and other drugs) to target and inhibit these key molecules. Many approaches seek to prevent infection, mitigate symptoms, and enhance treatment efficacy. Here, this review provides a synopsis of current research into the infection mechanisms of SARS-CoV-2 and its variants, alongside a review of recent global advancements in anti-SARS-CoV-2 drug development.

## 2. Structural Features and Interacting Receptors of SARS-CoV-2

**Figure 1:**
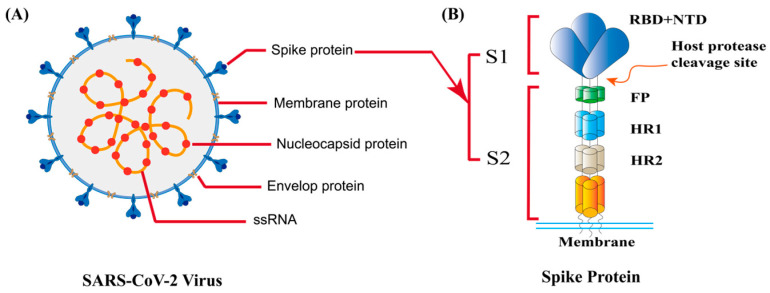
Structural features of SARS-CoV-2. (**A**). Virus structure of SARS-CoV-2; (**B**). Structure of S-protein.

SARS-CoV-2 contains a single-stranded RNA-enveloped virus with a genome size of approximately 30 kb. The genome encompasses about 4 structural proteins, 16 non-structural proteins, and 5–8 accessory proteins [7]. The primary structural proteins comprise the spike protein (S protein), envelope protein (E protein), membrane protein (M protein), and nucleocapsid protein (N protein) (Figure 1). The full length of the S protein spans approximately 1273–1285 amino acids [3], comprising two subunits: S1 and S2. The extracellular domain of the S1 subunit includes a conserved receptor-binding domain (RBD), facilitating the attachment of virus particles to the host receptor. The S2 subunit harbors a hydrophobic fusion peptide (FP) and two heptad repeat regions (HR1/2).

Following the binding of the S protein to the receptor, the HR1 and HR2 regions of the S2 subunit engage each other, forming a six-helix bundle. This interaction promotes the intimate association between the viral envelope and the cell or endosomal membrane, thereby facilitating membrane fusion [8]. The membrane protein (M) plays a crucial role in shaping the virus envelope, acting as the principal organizer for virus assembly. It works in concert with the nucleocapsid protein (N) and envelope protein (E) to collectively regulate the assembly, release, and infectivity of mature viruses.

ACE2 is a carboxypeptidase composed of 805 amino acids, pivotal in regulating essential physiological functions, such as cardiovascular activity and cellular inflammation, and potentially influencing the pathogenesis of central nervous system diseases [9,10,11]. It is extensively distributed on the surface of alveolar epithelial cells and small intestine cells and is found in the endothelial cells of arteries and veins, as well as arterial smooth muscle cells in all examined organs [12].

**Figure 2:**
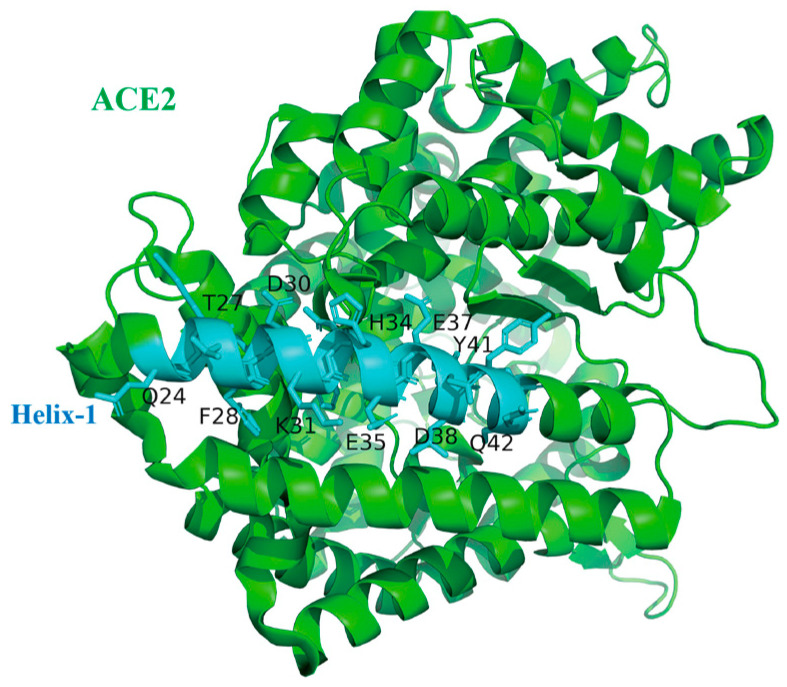
Structure of ACE2 (helix-1 in cyan, PBD ID:6M0J).

As a host receptor for SARS-CoV-2, numerous studies have elucidated the structural details of its interaction with the RBD of SARS-CoV-2 [13,14]. Among these, a long α-helix, referred to as helix-1, plays a crucial role in binding to RBD (Figure 2). Therefore, several studies have developed peptides that mimic this α-helix to inhibit RBD and have demonstrated initial success. However, in the design of the helical peptide, it is essential to consider and address how to maintain and reinforce the biologically active conformation of helix-1.

## 3. Invasion Mechanism of SARS-CoV-2

**Figure 3:**
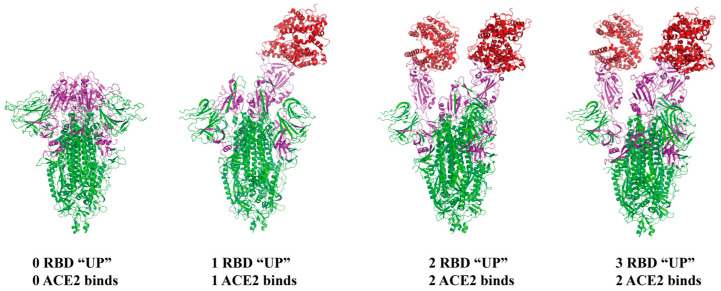
Structure of S protein binding ACE2. From left to right, the number of RBD in the upward conformation increases and binds more ACE2. The S protein is stained green, RBD is stained purple, and ACE2 is stained red. From left to right, PDB ID:7VRW, 7DX7, 7DX8, and 7DX9.

The S protein, serving as a pivotal apparatus for SARS-CoV-2 to infiltrate target cells, presents itself in a highly glycosylated homotrimeric configuration. Glycosylation plays a significant role in the appropriate folding of the S protein, its interaction with host proteinases, and its impact on the neutralizing efficacy of antibodies [15]. Under normal conditions, the states of the S protein RBD correspond to “up” (receptor-accessible or open) and “down” (receptor-inaccessible or closed) conformations (Figure 3).

The RBD can only bind to the hACE2 receptor and mediate membrane fusion between the virus and the cell when it is in the “up” conformation, exposed on the surface of the S protein, facilitating cell invasion [16]. Following attachment to the host cell surface, SARS-CoV-2 primarily enters the cell through two distinct pathways [17]. Both entry pathways hinge on host proteinase cleavage of the S protein at the junction of the S1 and S2 subunits, as well as at the S2’ site within the S2 subunit, subsequent to binding with the SARS-CoV-2 receptor. Initially, at the polybasic site (Arg-Arg-Ala-Arg) near the S1-S2 junction, Furin-like proteases, including proprotein convertases (PCs), recognize and cleave, resulting in the dissociation of S1 and S2 subunits and exposing the S2’ cleavage site. Subsequently, in the presence of transmembrane serine protease 2 (TMPRSS2), the S2’ site undergoes cleavage, exposing the fusion peptide (FP) to insert into the host cell membrane. The HR1 and HR2 regions of the S2 subunit undergo structural rearrangement through mutual recognition, forming a six-helix bundle.

This process facilitates the membrane fusion between the viral envelope and the host cell membrane [18]. If the host cell expresses insufficient TMPRSS2 or the virus-ACE2 complex fails to encounter TMPRSS2, the virus–ACE2 complex is internalized into endolysosomes via clathrin-mediated endocytosis. In this scenario, the cleavage of the S2’ site is executed by cathepsins within the endolysosomes [17]. Upon the onset of fusion between the virus and the cell membrane, a conformational shift occurs in the S protein, triggering the release of viral RNA into the host cell cytoplasm.

The 5’ end ORF1a and ORF1b regions are translated into two polyproteins, pp1a and pp1ab, which are subsequently cleaved into 16 non-structural proteins (Nsps). The 3’ end conservatively encodes structural proteins, including the spike (S) protein, envelope (E) protein, membrane (M) protein, and nucleocapsid (N) protein—four primary structural proteins along with some accessory proteins [8,19].

Subsequently, following replication and amplification of the viral genome by the RNA-dependent RNA polymerase (RdRp), diverse components assemble in the endoplasmic reticulum-Golgi intermediate compartment (ERGIC). This assembly culminates in the generation of new virus particles, which subsequently fuse with the cell membrane within secretory vesicles. Ultimately, the virus is liberated from the host cell through exocytosis [20].

## 4. Mutational Characteristics of SARS-CoV-2

Like other RNA viruses, SARS-CoV-2 has undergone notable genomic mutations while adapting to the host, particularly in the region encoding the S protein. These mutations, compared to wild-type (WT) strains, enhance their infectivity toward hosts and their capacity to evade immunity induced by vaccines [21]. Studies suggest that simultaneous infection with two distinct SARS-CoV-2 variants can occur in the same host [22]. Furthermore, individuals who have previously recovered from infections with earlier variants may remain susceptible to secondary infections with new variants, such as Omicron [23]. SARS-CoV-2 samples have been extensively isolated and sequenced by numerous laboratories worldwide. However, owing to its exceptional transmissibility, a significant number of viral genome mutation sequences have emerged globally. The majority of these mutations do not significantly alter the prevailing trends of strains in specific regions, thus not triggering new rounds of public health events. Nonetheless, as mutations continue to occur and accumulate, there is the potential for the virus’s transmissibility, virulence, or immune evasion capabilities to be further augmented. This presents substantial challenges for public health safety, clinical treatments, and vaccine development.

The WHO categorizes SARS-CoV-2 variants into two types: Variants of Concern (VOC) and Variants of Interest (VOI). To date, five variants have been identified as VOC: Alpha (B.1.1.7), Beta (B.1.351), Gamma (P.1), Delta (B.1.617.2), and Omicron (B.1.1.529). Diseases caused by these variants demonstrate markedly elevated transmission rates, and their clinical manifestations and immune responses might not be uniform [5,24]. During the early phases of the COVID-19 outbreak, the Alpha strain demonstrated significantly greater infectivity compared to the wild strain. In clinical studies, the Alpha strain entailed a higher risk of severe illness and death than the non-Alpha strain [25,26], which was also the case for the Beta, Gamma, and Delta strains [27]. Due to their higher infectivity and more severe clinical symptoms, they have received significant attention from researchers. In contrast, the Omicron strain exhibits relatively mild clinical manifestations. Although this might be attributed in part to the enhancement of the basic immunity within the population, it is characterized by high transmissibility and a significant risk of reinfection or breakthrough infection. Currently, several subvariants of Omicron have partially or completely evaded neutralizing immunity conferred by vaccines and monoclonal antibodies [28,29,30].

### 4.1. The Alpha (B.1.1.7) Variant

The B.1.1.7 variant [31,32], also known as the Alpha variant, was initially detected in the United Kingdom in September 2020 and swiftly emerged as the dominant strain in the country. The viral genome of the Alpha variant harbors a total of 17 mutations, with 9 of them found in the S protein (del 69H-70V, del 144Y, N501Y, A570D, D614G, P681H, T761I, S982A, and D1118H). Among these mutations, the del 69H-70V variant typically emerges subsequent to certain mutations that enhance the binding affinity of the S protein to the ACE2 receptor or confer immune evasion, such as N501Y, N439K, and Y453F [33]. It can also act as a compensatory mutation, countering mutations that enable immune evasion while diminishing its own infectivity [34]. The N501Y mutation results in heightened affinity of the spike protein for the ACE2 receptor. Previous studies have demonstrated that the binding affinity of N501Y-RBD to ACE2 is 10 times higher than that of the WT strain [35].

The D614G mutation is ubiquitous across all VOC and displays enhanced infectivity [36]. This could be attributed to the alteration of the RBD conformation by D614G, facilitating improved binding with ACE2. Studies indicate that in the D614 strain, 46% of RBDs are in the “up” conformation, whereas in the G614 strain, 82% of RBDs are in the “up” conformation [37]. This transition of the RBD to the “up” conformation illustrates the conformational impact of the D614G mutation on the RBD, making it easier for the RBD to bind with the ACE2 receptor and thus enhancing the infectivity of viral particles. Data indicates that the transmissibility of the Alpha variant has quickly outpaced that of previously existing variants of the novel coronavirus in the UK, establishing it as the predominant new variant in the country [31,38].

### 4.2. The Beta (B.1.351) Variant

The Beta variant was initially detected in South Africa in October 2020, characterized by nine mutations in the S protein (L18F, D80A, D215G, R246I, K417N, E484K, N501Y, D614G, and A701V) and three amino acid deletions (del L242-L244). Among these, three mutations (K417N, E484K, and N501Y) located in the RBD notably enhance the affinity of the S protein for ACE2 [39,40], leading to an elevated transmission risk and decreased neutralization by monoclonal antibody recovery serum [41].

### 4.3. The Gamma (P.1) Variant

The Gamma (P.1) variant, first identified in Brazil in December 2020, presents 12 amino acid residue mutations in the S protein, including L18F, T20N, P26S, D138Y, R190S, K417T, E484K, N501Y, D614G, H655Y, T1027I, and V1176F. Similar to the Beta (B.1.351) variant, three mutations situated in the RBD (K417N, E484K, and N501Y) enhance its transmissibility to 1.7 to 2.4 times that of the WT strain [42].

### 4.4. The Delta (B.1.617.2) Variant

The Delta (B.1.617.2) variant, first identified in India in December 2020, was responsible for the severe COVID-19 surge in India in April 2021. Delta carries a distinct set of mutations (T478K, P681R, and L452R), granting it exceptionally high transmissibility and the capacity to evade neutralizing antibodies in individuals previously infected or vaccinated [43,44]. Some studies suggest that the P681R mutation near the Furin cleavage site in the Delta variant may enhance Furin’s cleavage at the S1/S2 site, resulting in heightened fusion between viral particles and host cells [45,46]. Additionally, the L452R mutation not only enhances affinity for ACE2 but also boosts the virus’s replication capability, thereby increasing infectivity. This was demonstrated through the preparation of various S protein mutation pseudoviruses, infecting and expressing ACE2 and TMPRSS2 in HEK293 cells. The L452R mutation has been demonstrated to markedly enhance the infectivity of pseudoviruses compared to the parent S protein. Furthermore, experiments employing recombinant viruses to assess viral replication have indicated that viruses harboring the L452R mutation exhibit greater transmissibility and higher viral loads than those carrying the parent S protein [47].

### 4.5. The Omicron (B.1.1.529) Variant

On 24 November 2021, South Africa initially reported the B.1.1.529 variant to the World Health Organization (WHO). Given evidence suggesting detrimental alterations in the epidemiology of COVID-19 and a sharp surge in cases observed in South Africa, WHO designated B.1.1.529 as a VOC and dubbed it “Omicron”. Presently, Omicron (B.1.1.529) has emerged as a prominent VOC in numerous countries, with identified subvariants such as BA.1, BA.2, BA.3, BA.4, BA.5, and others.

In the S protein, Omicron exhibits 30 substitutions, 6 deletions, and 3 residue insertions. Since most SARS-CoV-2 vaccines and monoclonal antibodies are tailored against the WT S protein, certain mutations, particularly those involving the RBD, may affect the effectiveness of antibody neutralization. Multiple studies indicate a decrease or complete loss of neutralizing activity in vaccine sera against Omicron. The XBB variant, resulting from the BJ.1 (BA.2.10.1.1) and BM.1.1.1 (BA.2.75.3.1.1.1) recombination of the BA.2 lineage, is presently recognized for its most potent antibody evasion, even surpassing BA.5 [48]. Plasma from individuals vaccinated with a three-dose inactivated vaccine (CoronaVac) exhibits significant escape capability. Even individuals who received three doses of vaccines (BNT162b2, Pfizer–BioNTech, or mRNA-1273, Moderna) and subsequently recovered from infections caused by BA.1, BA.2, BA.5, and even BF.7 show weaker neutralizing titers against the latest XBB and other mutant strains [30].

JN.1 is the second-generation sub-branch of the Omicron BA.2.86 variant. Since November 2023, the WHO has elevated BA.2.86 from “variants under monitoring” (VUM) to VOI because of the swift proliferation of JN.1 among global epidemic strains. In a study involving 39 post-vaccination serum samples, neutralization titers were diminished by approximately 15-fold against XBB.1.5 and EG.5.1 and further decreased to 20-fold against BA.2.86 and JN.1 compared to the original B.1 strain. However, the reduction did not show a significant difference between BA.2.86 and JN.1 [49]. This leaves the cause of the rapid escalation in the transmission rate of JN.1 uncertain. Furthermore, convalescent plasma from individuals who had received three doses of CoronaVac and experienced breakthrough infections with BA.5 or BF.7 and subsequently were reinfected with XBB was utilized in a separate study to evaluate neutralization titers against BA.2.86 and JN.1 [50]. The study revealed that compared to BA.2.86, JN.1 exhibited significantly enhanced immune evasion ability. However, this enhancement came at the cost of decreased binding ability to human ACE2. This reduced binding capability might be the underlying reason why JN.1 developed resistance to human antibodies and gained a transmission advantage. However, infected patients showed mild or asymptomatic clinical symptoms [51]. This may suggest that Omicron and its subvariants may adopt a relatively mild mutation strategy to spread during the current dynamic evolution, that is, to evade the immunity of the vaccinated population as much as possible to infect as many people as possible, and the pathogenicity of the virus after infection is the price of the improved immune evasion ability.

## 5. Anti-SARS-CoV-2 Therapies

### 5.1. Vaccine Treatment

Establishing effective herd immunity through vaccination remains the primary and most efficacious strategy against the virus. Currently, SARS-CoV-2 vaccines mainly encompass RNA vaccines, recombinant vaccines, viral vector vaccines, and inactivated vaccines. Apart from inactivated vaccines, the majority of vaccines mainly introduce the original or modified fragments from the viral spike as antigens and utilize the human immune system to recognize the epitopes of the target antigens and induce neutralizing antibodies, thereby generating long-lasting and specific humoral and cellular immunity. The Pfizer (BNT162b2) and Moderna (mRNA-1273) vaccines have been reported to provide 95% and 94.1% protection, respectively, in clinical patients early in the onset of COVID-19 [52,53].

Nevertheless, when the Omicron variant (B.1.1.529) initiated its pandemic spread, the spike protein, which serves as the vaccine antigen, undergoes numerous mutations, and the vaccine efficacy dropped to 65.5% (95% CI, 63.9 to 67.0) at 2 to 4 weeks following the second dose among patients who received 2 doses of BNT162b2. This declined to 15.4% (95% CI, 14.2 to 16.6) after 15 to 19 weeks and further dropped to 8.8% (95% CI, 7.0 to 10.5) after 25 weeks or longer. The effectiveness of the mRNA-1273 vaccine decreased from 75.1% (95% CI, 70.8 to 78.7) within 2 to 4 weeks to 14.9% (95% CI, 3.9 to 24.7) after 25 weeks or more [54]. Similarly, the AstraZeneca-Oxford vaccine (ChAdOx1 nCoV-19) showed no inhibitory activity against Omicron in serum from individuals 5 months after full vaccination [55]. Neutralization titers against Omicron were reduced or completely absent in sera from individuals inoculated with CoronaVac [54,56]. Thus, the protective efficacy of one or two vaccine doses is likely to be very limited, and a booster dose strategy for COVID-19 vaccines helps to reduce infection and severe disease [57,58].

As the Omicron subspecies JN.1 expedites its evolution toward immune evasion, it has the potential to trigger another pandemic at any moment, which might imply that the reduced effectiveness of vaccines or other drugs results from maternal and daughter mutations at certain sites. In the future, reasonable prediction of the changing trend of antigen mutation sites during the development of new vaccines or the development of broad-spectrum vaccines based on multiple antigens and obtaining a category of drugs targeting a specific lineage could be an effective approach to treat COVID-19 patients, yet it remains a formidable challenge. Therefore, the development and administration of vaccines need to remain a top priority, and further determination of whether effective agents have superior efficacy in the context of the current population immunity will contribute to effectively combating the new SARS-CoV-2 variants, which are associated with high rates of infection, virulence, pathogenicity, and mortality in the ongoing pandemic.

### 5.2. Advancements in Research on Drugs Targeting Virus Replication-Related Targets

#### 5.2.1. Inhibitors of RNA-Dependent RNA Polymerase (RdRp)

Remdesivir, Molnupiravir, Favipiravir, and other RdRp inhibitors have been commonly utilized in the treatment of COVID-19 in recent years Molnupiravir and Remdesivir exert their antiviral effects through distinct mechanisms. Remdesivir, Molnupiravir, Favipiravir, and other RdRp inhibitors have been widely employed in the treatment of COVID-19 in recent years [59,60]. Molnupiravir and Remdesivir exert their antiviral effects through distinct mechanisms. Remdesivir functions by acting as a substrate for the viral RNA chain elongation process utilized by RdRp, inducing elongation pause or termination [61]. Conversely, Molnupiravir induces widespread errors during the mutation introduction by RdRp, leading to premature translation termination and impeding virus replication [62]. The clinical efficacy of Remdesivir has been demonstrated in multiple randomized trials, making it the most widely employed drug. However, the clinical outcomes associated with Molnupiravir appear less promising. One clinical trial indicated that Molnupiravir did not reduce hospitalization or mortality rates when symptoms appeared in vaccinated adults with COVID-19 infection [63]. Another study similarly suggested that the clinical efficacy of Molnupiravir did not meet expectations [64]. Favipiravir, another RdRp-targeting drug, synergizes with Molnupiraviras, researchers found [65]. This combination increases the frequency of C-to-T mutations in viral RNA and reduces infectious virus titers in a hamster model compared to single treatment groups. Azvudine, developed by Henan Genuine Biotech Co., Ltd, Henan, China., is a novel antiviral drug. Studies have indicated that oral administration of Azvudine at 5 mg/day led to complete recovery for all treated COVID-19 patients, achieving a 100% rate of viral nucleic acid turning negative and discharge from medical care. However, mild dizziness and nausea symptoms may be associated with its use [66]. In a recent clinical study involving 804 non-hospitalized COVID-19 patients, the efficacy of Azvudine was evaluated. The findings demonstrated that Azvudine could reduce the progression speed and hospitalization rate among COVID-19 patients compared to the control group. Additionally, when taken within three days of symptom onset, it was observed to shorten the duration of fever [67]. Moreover, emerging monoclonal antibodies like CM12.1 [68], biomolecular proteins such as bovine lactoferrin (BLF) [69], compounds Aluminon and derivatives thereof [70], and phytochemicals derived from Withania somnifera [71] have demonstrated their ability to target RdRp. In particular, Aluminon, a cheap and common substance, also showed the ability to bind multiple residues of SARS-CoV-2 RBD in molecular modeling studies [72], making it a potential multitarget antiviral agent that holds promise as a next-generation treatment for COVID-19.

#### 5.2.2. Inhibitors of 3CL Protease

The 3C-like protease, a cysteine protease, cleaves pp1a and pp1ab proteins to release non-structural proteins NSP4 to NSP16, thereby participating in replication and transcription. It serves as one of the primary targets for antiviral therapy. Paxlovid, developed by Pfizer, is a combination antiviral drug comprising Nirmatrelvir and Ritonavir. In a phase II–III double-blind, randomized, controlled trial (NCT04960202) involving 2246 non-hospitalized adult patients treated within three days of symptom onset, the incidence of hospitalization or death was reduced by 89% [73]. However, recent clinical data involving critically ill adult patients with COVID-19 indicate that Paxlovid did not significantly improve the 28-day all-cause mortality rate or viral RNA clearance rate [74]. This could be attributed to the diminished immune response observed in severe adult patients, posing a challenge for Paxlovid. Despite its ability to inhibit the 3CL protease and impede viral replication, clearing viral RNA through the natural immune response may be compromised. Another study unveiled a novel non-covalent 3CL protease inhibitor, Ensitrelvir (S-217622), exhibiting an in vitro EC_50_ range of 0.29 to 0.5 μM against SARS-CoV-2 VOC variants [75]. Its extended in vivo elimination half-life and excellent oral bioavailability indicate its potential as a candidate oral drug for clinical treatment of COVID-19. In a clinical study encompassing 341 subjects, treatment with Ensitrelvir significantly reduced the viral titers and RNA concentrations of the Omicron variant in patients with mild to moderate COVID-19, with no reported severe side effects [76]. However, the study had a limited number of elderly patients, and the risk of severe COVID-19 increases with age. Additionally, symptoms of Omicron strain infection are generally milder. More extensive phase III clinical trials are currently underway. EDP-235 is an effective selective inhibitor of 3CL protease, demonstrating inhibitory activity against SARS-CoV-2 and several VOC of 3CL protease (IC_50_ = 2.8–5.8 nM) [77]. The protease activity of 3CLpro demonstrates a significant inhibition curve in both time and concentration-dependent manners, indicating that EDP-235 may function as a slow-acting, mild inhibitor. This characteristic could potentially offer an advantage over other inhibitors.

#### 5.2.3. Inhibitors of Papain-like Protease (PLpro)

PLpro, a cysteine protease, cleaves pp1a and pp1ab proteins to release NSP1, NSP2, and NSP3. Additionally, it removes host ubiquitin and ubiquitin-like interferon-stimulated gene 15 (ISG15) from signaling proteins, inhibiting the innate immune response [78]. F0213, a PLpro inhibitor [79], demonstrates antiviral activity against Alpha, Delta, Omicron, and MERS-CoV. On one hand, F0213 can inhibit PLpro to counter virus-induced immune imbalance. On the other hand, it can suppress coronavirus replication by blocking the cleavage of viral pp1a and pp1ab polyproteins. Acriflavine (ACF) is an affordable drug approved in certain countries. Researchers [80] discovered that, through high-throughput screening of PLpro inhibitors, ACF broadly inhibits the replication of SARS-CoV-2 and other beta coronaviruses in cellular models at nanomolar concentrations. In a mouse model, oral administration of 100 mg/kg or intramuscular injection of 5 mg/kg ACF completely blocked SARS-CoV-2 infection in the mouse brain. However, due to the relatively short half-life of ACF in the body [81], it may be more suitable for short-term treatment. Combining it with other drugs could potentially compensate for its short half-life. Additionally, GRL0617, initially identified as a PLpro inhibitor against SARS-CoV [82], has been found in the current study to act as a non-covalent competitive inhibitor of SARS-CoV-2 PLpro. It demonstrates significant inhibition of SARS-CoV-2 virus replication in Vero E6 cells in a dose-dependent manner [83]. At a concentration of 100 μM, the inhibitory activity exceeded 50% without significant observed cytotoxicity. Furthermore, the residues involved in the interaction between SARS-CoV and SARS-CoV-2 with PLpro are conserved, and the interaction with all compounds is also conservative. The anticipated ability of GRL-0617 to inhibit SARS-CoV-2 virus replication provides compelling evidence for further exploration of “repurposing old drugs” strategies.

### 5.3. Advances in Research on Drugs Targeting ACE2

The host cell receptor ACE2 is a membrane-bound peptidase expressed in various tissues, including the heart, lungs, kidneys, and epithelial cells lining blood vessels and the lower digestive tract [12]. Due to its high affinity with the RBD region of SARS-CoV-2 and minimal mutation risks, targeting ACE2 has emerged as a prominent research focus for inhibiting SARS-CoV-2 infection. Chloroquine (CQ) and hydroxychloroquine (HCQ), long used for malaria treatment, have been found to inhibit SARS-CoV entry by altering glycosylation of the ACE2 receptor and the spike S protein, as demonstrated in previous studies against SARS-CoV [84]. In the treatment of COVID-19, CQ effectively inhibits SARS-CoV-2 in vitro (EC_90_ = 6.90 μM) [85] and was included in the experimental drug list of the “COVID-19 Diagnosis and Treatment Guidelines (Sixth Edition)” issued by the National Health Commission of the People’s Republic of China. However, subsequent clinical trials revealed that CQ and HCQ did not achieve expected preventive effects compared to the placebo and may even lead to more severe side effects [86,87].

In addition, recent studies have found that bile acids regulate human ACE2 levels through the Farnesoid X receptor (FXR), offering a potential avenue for preventing SARS-CoV-2 infection by modulating ACE2 expression levels. Experimental data demonstrate that FXR-mediated reduction in ACE2 expression levels decreases the susceptibility of various cells to SARS-CoV-2 in vitro. Similarly, experimental results from mouse and human lung models indicate that treatment with ursodeoxycholic acid (UDCA), which regulates FXR signaling in mice or human lungs, not only reduces ACE2 expression but also mitigates infection symptoms in mice while lowering infection risk in human lungs [88]. However, further experiments or clinical data are required to elucidate the efficacy and safety aspects associated with this approach. Another potential strategy involves utilizing soluble ACE2 or ACE2-mimicking peptides for prevention against SARS-CoV-2 [89,90]. Nonetheless, in a Phase II trial involving hospitalized COVID-19 patients (NCT04335136), APN01 (recombinant human angiotensin-converting enzyme 2, rhACE2) did not demonstrate a significant benefit in terms of the 28-day all-cause mortality or improvement in clinical symptoms compared to the placebo control group. However, a recent study suggests that the small molecule SB27041 can target a conformational binding site in the ACE2 structure, potentially blocking its interaction with the SARS-CoV-2 S protein by inducing changes in its conformation. Simultaneously, it preserves the physiological activity of ACE2, showcasing significant clinical potential for small molecule drugs [91].

### 5.4. Research Progress on Drugs Targeting Proteases

The invasion of SARS-CoV-2 into cells relies on host proteases such as Furin, Transmembrane Serine Protease 2 (TMPRSS2), Cathepsin, and others that facilitate cleavage or membrane fusion. Therefore, targeting these proteases holds promise for the development of clinical drugs against SARS-CoV-2.

#### 5.4.1. Furin Inhibitors

The Furin cleavage site plays a pivotal role in the evolution of SARS-CoV-2, with P681H/R emerging as a recent hotspot for mutations. This mutation is frequently observed in variants such as Alpha, Delta, Omicron, and their derivatives. These mutations could potentially accelerate Furin cleavage at the S1/S2 site, thereby possibly boosting infectivity compared to the wild-type SARS-CoV-2. Earlier studies assessed two oral (BOS-981 and BOS-318) and one inhaled (BOS-857) Flynn inhibitors [92]. The findings showed that BOS inhibitors efficiently hindered the S1/S2 cleavage of soluble Furin enzyme (IC_50_ = 8.8~9.4 nM). Furthermore, complete suppression of SARS-CoV-2 infection in lung-derived Calu-3 cells was achieved through the combined inhibition of Furin enzyme (BOS) and the type II transmembrane serine protease 2 (TMPRSS2) inhibitor (Camostat). A synthetic peptide mimic, MI-1851, at a concentration of 10 μM, exhibited inhibition of the Furin enzyme in human lung cancer Calu-3 cells, resulting in the prevention of SARS-CoV-2 spread, and a reduction in virus titer by up to 75 times. Complete blockade of SARS-CoV-2 replication was observed when MI-1851 was used in combination with T-ex5 PPMO (TMPRSS2 inhibitor) [93]. Another study suggested that MI-1851 appeared to have no detrimental effects on the viability of primary human liver cells [94], providing important safety data for the strategy of inhibiting the Furin enzyme to counteract SARS-CoV-2.

#### 5.4.2. TMPRSS2 Inhibitors

Camostat mesylate and Nafamostat mesylate are two serine protease inhibitors that have demonstrated efficacy in blocking SARS-CoV-2 membrane fusion by targeting TMPRSS2 [18,95]. However, two independent, double-blind, randomized, placebo-controlled clinical trials demonstrated that Camostat mesylate did not improve symptoms in hospitalized adults with COVID-19 (NCT04657497, NCT04321096) [96,97]. Another Phase II open-label, randomized controlled trial consistently reported no significant differences between Nafamostat mesylate and the control group in mild COVID-19 patients, with only limited efficacy observed in a small subset of high-risk COVID-19 patients (NCT04623021) [98]. Researchers have also discovered a small molecule compound, N-0385, which exhibits low nanomolar potency in inhibiting TMPRSS2, thereby blocking SARS-CoV-2 infection (EC50 = 2.8 nM) [99]. N-0385 demonstrated strong inhibitory activity against four VOCs (Alpha, Beta, Gamma, and Delta) within a low nanomolar range. In a mouse experimental model, N-0385 significantly increased the survival rate of SARS-CoV-2-infected mice compared to the control group, reaching 70%. It also reduced lung alveolar edema, alveolar fibrosis, and the presence of inflammatory cells in the alveoli, along with antigen and viral titers in the tissues. This indicates the potential of N-0385 as a countermeasure against newly emerging volatile variants of SARS-CoV-2. Recently, researchers identified a novel TMPRSS2 inhibitor, BC-11, based on computational modeling and in vitro enzyme assays [100]. Pseudovirus (PsV) experimental results showed that BC-11 exhibited in vitro inhibitory activity against the Omicron variant (EC_50_ = 66 μM), although with lower efficiency compared to Camostat (EC_50_ = 0.98 μM). Combining drugs could be one approach to enhance their inhibitory activity.

#### 5.4.3. Cathepsin Inhibitors

It was previously noted that SARS-CoV-2 can enter cells through two pathways: the endocytic pathway with Cathepsin and the membrane fusion pathway with TMPRSS2. In the context of the global spread of the Omicron variant, studies indicate that the efficiency of Omicron in entering cells through the endocytic pathway with host cell proteases has increased by more than fourfold, while the efficiency through the TMPRSS2 pathway has decreased by more than threefold [101].

Therefore, protease inhibitors targeting host cell proteases may be more effective in preventing the entry of the Omicron variant into cells. Teicoplanin, a glycopeptide antibiotic, has been shown to inhibit Cathepsin L effectively and can inhibit PsV infections of Ebola, MERS-CoV, and SARS-CoV [102]. Similarly, experimental results showed that Teicoplanin, with a half-maximal inhibitory concentration (IC_50_) lower than 5 μM for most viruses, could prevent SARS-CoV-2 infection in hACE2 mice [103]. In clinical settings, the serum concentration of Teicoplanin is at least 15 mg/L (8.78 μM) after completing the therapeutic dose for most Gram-positive bacterial infections [102]. This suggests that Teicoplanin, used for clinical treatment of SARS-CoV-2, not only demonstrates safety but also indicates its potential as a dual inhibitor for SARS-CoV-2 and Gram-positive bacterial co-infections. E-64d is a broad-spectrum, low-toxicity Cathepsin inhibitor that can inhibit CatB, CatL, CatK, and CatH. In vitro experiments, E-64d significantly inhibited the entry of WT, D614G, N501Y, and Delta S protein pseudoviruses [104]. Camostat mesylate, the control, was insensitive to PsV entry, indicating that Cathepsin and TMPRSS2 correspond to two different invasion pathways of SARS-CoV-2. It is worth noting that most protease inhibitors, such as MPI8 [105], MDL28170 [106], and MG132 [107], have only been identified in vitro PsV experiments with the ability to resist SARS-CoV-2, and further evaluation of more variants and clinical effects is needed. However, with the prevalence of Omicron, the Cathepsin-mediated endocytic pathway has become dominant. Combining Cathepsin inhibitors with other targets may offer more advantages for preventing and treating the current situation.

#### 5.4.4. Advancements in Research on Inhibitors Targeting Other Host Enzymes

Other enzymes indirectly implicated in virus–cell binding could serve as potential targets, in addition to the proteases mentioned earlier. PIKfyve, a phosphoinositide kinase, plays a role in phosphorylating PtdIns3P to PtdIns(3,5)P2, thus regulating membrane homeostasis. Apilimod, an established inhibitor of PIKfyve, demonstrates potent inhibitory effects in preventing the entry of various SARS-CoV-2 variants into host cells [108]. Recent research discovered that another PIKfyve inhibitor, XMU-MP-7, achieved complete inhibition in vitro against WT SARS-CoV-2 and four VOCs (Alpha, Beta, Delta, and Omicron) at a concentration of 200 nM. Moreover, both XMU-MP-7 and Apilimod mitigated the cytopathic effects induced by SARS-CoV-2 and its Omicron variant. This can be attributed to their dual action of impeding virus membrane fusion, hindering the release of viral RNA into the cytoplasm, and inhibiting proteases. Caspase, a cysteine-aspartic protease, regulates the cascade reaction leading to host cell apoptosis [109]. Researchers observed significant inhibitory effects on MERS-CoV and SARS-CoV-2 when employing z-VEID-fmk as a caspase inhibitor in human ex vivo lung tissues, human intestinal organoids, and animal models. Treatment with z-VEID-fmk improved various types of tissue damage, including bronchiolitis, alveolitis, and vasculitis, in the lungs of treated hamsters. The mechanism behind this may involve coronaviruses interfering with host IFN signaling through caspase-6-mediated N protein cleavage to facilitate efficient replication, thus weakening viral replication capacity upon caspase-6 inhibition [110]. Furthermore, Bromo-domain-containing protein 2 (Brd2) [111], TMEM16 [112], GSK-3 [113], and others have been identified as potential therapeutic targets. Nonetheless, the majority of drugs targeting these entities are presently in the preclinical development stage and necessitate further clinical validation.

### 5.5. Advancements in Research on Drugs Targeting the Spike Protein

#### 5.5.1. Neutralizing Antibodies Targeting S1

Antibodies have been extensively employed to neutralize viral antigens in the treatment of viral diseases and have been a central focus in COVID-19 drug research. Presently, the primary target for neutralizing antibodies is the RBD on the Spike protein of the SARS-CoV-2 virus particle. These antibodies exert a blocking effect by restricting the virus from entering host cells. Evusheld (tixagevimab + cilgavimab; AZD7442) is a combination antibody drug that targets the spike protein. It is not only utilized for clinical treatment but has also received approval from the U.S. FDA for pre-exposure prevention. In the PROVENT Phase III pre-exposure prevention trial, Levin and colleagues randomly assigned 5197 participants to receive a single dose of Evusheld (150 mg tixagevimab + 150 mg cilgavimab; *n* = 3460) or a placebo (*n* = 1737) [114]. The results indicate that compared to the placebo, the group receiving a single dose of Evusheld (150 mg tixagevimab + 150 mg cilgavimab) reduced the risk of symptomatic COVID-19 infection by 82.8% (95% CI: 65.8 to 91.4). Among patients treated with Evusheld, there were no cases of severe symptoms or deaths, while in the placebo group, there were five severe cases, including two deaths. Additionally, Evusheld possesses a longer half-life and demonstrates resistance to immune escape.

Studies have demonstrated that Evusheld retains neutralizing activity against the Alpha, Beta, Gamma, and Delta variants and may provide protection for up to 12 months [115]. However, in studies targeting Omicron, Evusheld has shown a reduction in neutralizing activity against it compared to other VOC [116]. Despite this, research has indicated that although the neutralizing activity of Evusheld against Omicron is diminished, it still reduces viral load and lung inflammation in mice [117]. This suggests that, aside from retaining some neutralizing activity against Omicron, it may also contribute to alleviating certain clinical symptoms, although this has not been confirmed in clinical settings.

Amuvatinib and Remcivir monoclonal antibodies (BRII-196/BRII-198) are monoclonal neutralizing antibodies developed through a collaboration between Tengshengbo Pharmaceutical, Shenzhen Third People’s Hospital, and Tsinghua University. In vitro PsV data indicate that Amuvatinib and Remcivir monoclonal antibodies retain neutralizing activity against major SARS-CoV-2 variants, including Alpha, Beta, Gamma, Delta, as well as BA.1, BA.1.1, and BA.2 [118,119]. However, in a randomized, double-blind clinical trial (NCT04501978) targeting adults hospitalized for COVID-19 without organ failure and with symptoms lasting up to 12 days, treatment with BRII-196+BRII-198 showed no significant difference compared to a placebo. There was no apparent benefit observed in improving the lung status on the fifth day, clinical recovery time, or other clinical outcomes for patients [120].

Bebtelovimab is a monoclonal antibody drug developed by Eli Lilly. In vitro virus neutralization studies indicate that Bebtelovimab maintains effective neutralizing activity against various variants, including B.1.1.7, B.1.351, B.1.617.2, B.1.427/B.1.429, P.1, B.1.526, B.1.1.529, and the BA.2 subvariant. It also retains binding to the S protein with multiple potential RBD mutations, including K417N, L452R, E484K, and N501Y [121,122]. The broad and effective neutralizing activity, along with the relatively conserved epitope, suggests its potential as an effective antibody drug for treating COVID-19.

The N-terminal domain (NTD) is a significant target for antibodies, alongside the RBD on S1. Although it is not highly immunogenic, early studies have identified many monoclonal antibodies that recognize this structure and have effectively neutralized SARS-CoV-2 infection in vitro, suggesting its potential as a clinical therapy strategy [123,124,125]. Recent studies have shown that NTD-targeted neutralizing antibodies are effective against SARS-CoV-2 mutations. For instance, SA-3 can bind to the N4 loop on NTD (EC_50_ = 16 ± 2 nM) and exhibit strong neutralizing activity against BA.1 in vitro (NT_50_ = 57 ± 17 nM), compensating for the reduced potency of RBD antibodies against mutants when used in synergy with S-E6, an antibody targeting RBD [126]. Similarly, combining NTD and RBD antibodies has been reported to have a more significant effect in inhibiting the efficacy of mutants [127,128].

For various reasons, the development of broadly neutralizing antibodies against SARS-CoV-2 variants remains challenging. On one hand, the spike protein of SARS-CoV-2 is highly variable, making the emergence of resistant mutations likely [129]. Most existing anti-S protein antibodies have shown reduced or completely diminished neutralizing activity against notable Omicron variants. On the other hand, serum titers of anti-SARS-CoV-2 antibodies decrease over time, and most antibodies may provide protection for only a few months [130].

#### 5.5.2. Neutralizing Reagents Targeting S2

Generally, SARS-CoV-2 typically enters host cells through two pathways: fusion either at the plasma membrane or within endosomes. The HR1 and HR2 domains of the S2 subunit play a pivotal role in mediating virus–cell membrane fusion, making them promising targets for inhibiting viral infection.

COV44-62 and COV44-79 are monoclonal antibodies that target the conserved fusion peptide (FP) region. Studies have demonstrated their ability to neutralize not only the WT SARS-CoV-2 but also notable variants, including Omicron BA.2 and BA.4/5 subvariants [131]. The NT_50_ for COV44-62 against Omicron BA.1, BA.2, and BA.4/5 ranges from 10.38 to 51.89 μg/mL, while for COV44-79, it ranges from 33.02 to 55.44 μg/mL. In mouse models, both antibodies were found to reduce weight loss and improve recovery rates compared to control mice (*p* < 0.01, days 3–7 for COV44-79; *p* < 0.05, days 5–7 for COV44-62).

Furthermore, COV44-79 showed some enhancement in lung virus titers and pulmonary symptoms in mice. EK1 is a pan-coronavirus membrane fusion inhibitory peptide that targets the HR1 domain of the HCoV S protein by disrupting the interaction between HR1 and HR2 during the formation of the six-helix bundle (6-HB). Researchers have found that EK1C4, synthesized by coupling cholesterol molecules with the EK1 peptide, displayed highly effective inhibitory activity against SARS-CoV-2-mediated membrane fusion and PsV infection during the early stages of the outbreak (IC_50_ = 1.3 nmol/L for cell-cell fusion; IC_50_ = 15.8 nmol/L for PsV) [132]. In their recent study, the team found that EK1, EK1C4, and EKL1C retained inhibitory activity against Omicron PsV, with IC_50_ values of 309.4 nM, 8.63 nM, and 26.14 nM, respectively [133], possibly due to the relatively conserved nature of the S2 subunit. Similarly, another study reported that both membrane fusion inhibitors, dimeric and monomeric SARS-CoV-2 HRC lipopeptides, effectively suppressed membrane fusion mediated by the S protein of all VOCs. The BA.1 S protein of Omicron was the most sensitive to inhibition, with an IC_50_ of approximately 0.2 nM inhibited by the SARS-CoV-2 HRC dimer.

In contrast, serum from vaccinated individuals and commercial monoclonal antibody preparations lost neutralizing potency against BA.1 and BA.2, suggesting that fusion inhibitory peptides could emerge as a treatment strategy unaffected by viral evolution [134].

## 6. Conclusions

In recent years, with the outbreak and global pandemic of SARS-CoV-2 infection, many researchers have directed their efforts toward understanding the infection mechanism of SARS-CoV-2, thus identifying potential targets for the development of drugs to combat COVID-19. Building on this foundation, considerable global progress has been achieved in developing promising antiviral drugs. However, effective treatments against SARS-CoV-2 remain elusive. The emergence of Omicron has rekindled a major outbreak of SARS-CoV-2 and underscored the ineffectiveness of current vaccines, emphasizing the urgency of combating the activity of SARS-CoV-2 and its variants. The research and development of COVID-19 vaccines has been continuing worldwide, but we still need to face up to some shortcomings. Specifically, the different effectiveness of vaccines in different populations, the rapid mutation rate of the COVID-19 virus, the inability of a single vaccine to provide long-term protection, and the possible side effects after vaccination in some recipients are all problems that need to be solved urgently. For example, vaccine efficacy in older adults and those with weakened immune systems may not be as dramatic as in younger healthy individuals, which not only presents a challenge in vaccine rollout but also raises concerns about the development of herd immunity. Therefore, the development of more targeted broad-spectrum vaccines and the optimization of vaccination strategies for specific populations will be one of the focuses of future efforts.

The newly emerged Omicron variant continues to undergo continuous evolution, demonstrating high resistance to neutralizing antibodies induced by current COVID-19 vaccines, convalescent sera, and therapeutic monoclonal antibodies. The neutralizing activity of these drugs also diminishes over time. This underscores the need for the development of highly effective anti-COVID-19 drugs with characteristics, such as resistance to mutations, prolonged duration of action, and high safety profiles, in order to keep pace with the emergence of new variants. Recent studies indicate that the Omicron variant is highly sensitive to membrane fusion inhibitors targeting the S2 subunit HR1 domain and enzyme inhibitors responsible for intracellular membrane fusion. This sensitivity may be related to its preference for the entry pathway, offering hope for breakthroughs in the development of drugs against the Omicron variant and other variants in the future. Meanwhile, drugs capable of simultaneously targeting multiple pathways and employing synergistic combinations have shown promising outcomes in several studies. Cocktail therapy has proven effective in treating various diseases. Presently, single-drug clinical interventions often yield limited protective effects or prove ineffective. To specifically target preferential mutations observed in Omicron variants, multi-targeted drugs may emerge as strong candidates for future COVID-19 treatments.

## Data Availability

Not applicable.
